# Audience effects in sooty mangabey agonistic behavior

**DOI:** 10.3389/fpsyg.2025.1551210

**Published:** 2025-09-30

**Authors:** Fredy Quintero, Sonia Touitou, Martina Magris, Klaus Zuberbühler

**Affiliations:** ^1^Department of Comparative Cognition, Institute of Biology, Université de Neuchâtel, Neuchâtel, Switzerland; ^2^Centre Suisse de Recherches Scientifiques, Taï Monkey Project, Abidjan, Côte d'Ivoire; ^3^School of Psychology & Neurosciences, University of St Andrews, St Andrews, United Kingdom

**Keywords:** *Cercocebus atys*, rank, audience effects, aggression, social cognition, communication, social awareness

## Abstract

The term ‘Audience Effects’, refers to behavioral changes triggered by the mere presence of others and has been extensively studied in animals to explore their capacity for social awareness and intentionality. Research shows that a wide range of species—from insects to primates—alter behaviors depending on their audience, with primates, especially great apes, demonstrating the most complex audience-aware behaviors, such as adjusting communication based on the recipient’s attention or understanding. These findings suggest that some animals can infer intentions, remember social dynamics, and strategically act depending on who is watching. However, there is still limited data from non-ape primates and other mammals, raising questions about whether such cognitive traits evolved through shared ancestry or convergent evolution. Aggressive behaviors also reveal audience effects, with individuals, especially lower-ranking ones, using strategic aggression in front of influential bystanders to influence future interactions. In this study, we used focal animal sampling to investigate how free-ranging sooty mangabeys, a terrestrial forest-dwelling primate living in large groups, used aggression depending on the composition of the audience. We found that individuals were significantly more aggressive to opponents if they were observed by large audiences that contained higher ranking individuals. These displays of aggression were often accompanied by vocalizations, further suggesting that aggressors were interested in attracting the audience’s attention. We discuss these patterns of audience-dependent aggressive behavior and propose that sooty mangabeys adjust their social behavior depending on the composition of the bystanding audience, reacting in the most appropriate way depending on the situation, which provides additional support to the growing body of research showing that the underlying mechanisms necessary for the evolution of complex social cognition are more widespread in the animal kingdom than was previously thought.

## Introduction

The term ‘Audience Effect’ comes from human psychology studies from more than 100 years ago (Triplett, 1898) and it is defined as the change on a subject’s behavior by the mere presence of someone else (the audience) (Coppinger et al., 2017). They have been the subject of many comparative studies on animals due to the possibility of intentionality; animals might change their behaviors strategically depending on who’s in the audience (Zuberbühler, 2008). There are many reasons why this is important, but to put it simple, how aware are animals of other animals is one of the biggest differences between them and humans, the consensus is that great apes can perceive and attribute intentions to others. They can also communicate their own intentions and, if misunderstood or ignored, modify their signaling strategy to achieve their goal (Call and Tomasello, 2008; Krupenye et al., 2016; Townrow and Krupenye, 2025). Thus, understanding how the audience is driving the occurrence of certain behaviors in animals will shed light on the underlying mechanisms that led to the development of the human-mind like behaviors.

Research on audience effects is well documented in the animal kingdom. A first major finding is that all kinds of animals, from insects to mammals, can be affected by the presence of others (Cheney and Seyfarth, 1990; Marler et al., 1986; Matos et al., 2003; Pollick et al., 2005; Ridley et al., 2007; Sherman, 1977; Zajonc et al., 1969). Most studies on audience effects in animals have been focused on how certain audiences affect an individual’s calling rate and composition (Slocombe and Zuberbühler, 2007). This focus on signaling behaviors may be due to the fact that they are easy to measure, and can be replicated and manipulated to simulate the presence of certain audiences, both in captivity and in the wild.

Overall, natural selection is expected to favor animals that are able to adjust their behaviors to maximize their own reproductive success, by taking into account characteristics of their audiences, such as composition and attention (Zuberbühler, 2008). Such audience awareness is likely to be found in species where individuals attend and learn from watching the social interactions between the members of their group. Here, compelling evidence is from primates, birds and fish that can deduce, for example, the dominance relations of other individuals by just looking at the outcome of conflicts (Bond et al., 2003; Davis, 1992; Gillan, 1981; Grosenick et al., 2007; Lazareva et al., 2004; McGonigle and Chalmers, 1977; Rapp et al., 1996; Roberts and Phelps, 1994; Steirn et al., 1995; Von Fersen et al., 1991).

For non-human primates, the evidence suggests that individuals can take into account the goals and intentions of others and adjust their own behaviors accordingly (Call and Tomasello, 2007; Hare, 2011). For example, studies with captive orangutans, chimpanzees and bonobos have all shown that individuals are capable of modifying their signal output depending on the attentional state and familiarity of the recipient, with evidence for persistence and elaboration when dealing with unresponsive recipients (Cartmill and Byrne, 2007, 2010; Genty et al., 2015; Hobaiter and Byrne, 2014). A number of great ape field studies have also concluded that some vocal behavior meets key criteria for intentionality (e.g., chimpanzees: Crockford et al., 2012; Hobaiter et al., 2014; Schel et al., 2013; bonobos: Genty and Zuberbühler, 2014). One challenging hypothesis from this research is that great apes are not only able to perceive and attribute intentions to others, but that they are also able to communicate their own and, if misunderstood or ignored, modify their signaling strategy to achieve the desired goal (Zuberbühler, 2008).

As remarked above, the main advances have come from great ape research while comparably less is known from other groups of primates and non-primate mammals. This is problematic for evolutionary theories of cognition, for example, whether audience awareness evolves along phylogenetic lines, perhaps as mere by-products of increasingly large brains or whether it can evolve by convergent evolution in response to specific socio-ecological challenges (Emery and Clayton, 2004). To address these questions, research on monkeys and non-primate species is crucial.

Here, some relevant findings come from captive rhesus macaques (*Macaca mulatta*) and tufted capuchins (*Sapajus apella*), which show that subjects can be sensitive to others’ goals and intentions (e.g., Drayton and Santos, 2014; Flombaum and Santos, 2005; Hare et al., 2003; Phillips et al., 2009; Santos et al., 2006). In the wild, there is also evidence showing that some monkey species are able to produce alarm calls with the apparent purpose of influencing others’ behaviors (Zuberbühler, 2018). In one study, wild Thomas langur males continued to produce alarm calls to predator model until every group member had responded with at least one alarm call, as if to ensure that others were aware of the danger (Wich and de Vries, 2006). In another study, wild Diana monkey females continued to alarm call until their own male produced the semantically matching (‘correct’) alarm calls, i.e., the predator spotted by the females, in response to which they stopped producing alarm calls (Stephan and Zuberbühler, 2016). Also, playback experiments with blue monkeys showed that males produced significantly more alarm calls to simulations of crowned eagle presence if other group members were closer to the presumed predator than far away (Papworth et al., 2008), further demonstrating some basic audience awareness, but not ruling out explanations based on basic changes in affective states.

Overall, the evidence suggests that apes, and some other primates, do more than just evaluate their audience in terms of biologically important categories; they also take into account psychological variables, such as attention, ability to comprehend (Call and Tomasello, 2007; Crockford et al., 2012) and capacity to help (Slocombe and Zuberbühler, 2007). However, it is not clear if this is a general feature of primate cognition or limited to some species and behavioral contexts. Although research on great apes continues to provide evidence for audience effects and intentional communication (e.g., Bouchard and Zuberbühler, 2022; Gruber and Zuberbühler, 2013; Schel et al., 2013), to our knowledge, there are no comparable studies on free-ranging monkeys designed to tackle the same questions. Studying cognition in the wild is generally difficult, suggesting that no single experiment will be powerful enough to provide conclusive answers.

One group of behaviors that would require certain audience awareness and cognitive resources that may not be available to every species, because of the time dimension, social complexity and the corresponding long-term memory load, are the aggressive behaviors (Santos et al., 2021). This is relevant for species in which bystanders intervene in ongoing conflicts, either in support of the victim or, more commonly, the aggressor (Petit and Thierry, 1994; Schino, 2007). Being aggressive and whether to intervene becomes part of an equation based on past events, current dispositions and future consequences for which keeping track of third-party relationships (i.e., who will support whom) is key.

There are interesting species differences in terms of what kind of support individuals can expect from their audiences. In vervet monkeys, for example, bystanders largely prefer to support aggressors (Mercier et al., 2019), whereas in chimpanzees, victims can get help from bystanders, which has led to the finding that victims sometimes use vocalizations strategically (Slocombe and Zuberbühler, 2007). But even if bystanders do not interfere, they will usually take note of the nature and outcome of an ongoing conflict. This is especially important for lower-ranking individuals, who may decide to engage in aggression if this is observed and remembered by uninvolved bystanders. For example, being aggressive in front of high-ranking observers may inform them of one’s fighting ability or readiness to escalate (Arnott and Elwood, 2009; Martín and López, 2007; Yasuda and Koga, 2016) and, as a consequence, result in future tolerance from them. To summarize, being aggressive in front of uninvolved higher-ranking bystanders is one solution for how low-ranking individuals can avoid harassment from higher-ranking individuals, but this requires a minimum degree of audience awareness.

There is substantial literature on audience effects in aggressive situations, but mostly from non-primate species and without much evidence for complex decision-making. For instance, cichlid fish experience changes in androgen levels after watching fights (Oliveira et al., 2001), male red-bellied woodpeckers reduce displays to other males and increase social vocalizations when females arrive (Miles and Fuxjager, 2019) or male fiddler crabs are more aggressive to intruders if they have witnessed aggression before (Darden et al., 2019). The focus of our study was different insofar as we were interested in whether animals increased their aggressive behavior when watched by others, not before and after the arrival of certain ‘audience’ individuals, nor before and after the occurrence of a determined event, but in average in the presence of specific bystanders. In order to keep it simple for this study, we defined audience effects as the change in the behavior of the ‘approacher’ (the individual that has the intention of interacting with another) toward the ‘approached’ (the individual who is target of the approacher’s behavior), by the presence of at least another individual that is not part of the interaction.

To this end, we aimed to test whether sooty mangabeys, a highly social and generally tolerant forest monkey (Range et al., 2007), were capable of modifying their aggressive tendencies when locked into competitive interactions with other group members, depending on the audience composition. In forest habitats with limited visibility, audience compositions change all the time, suggesting that forest primates, on which social structure, personal space and group composition are of paramount importance, need to keep track of who is able to observe them and what their likely response will be (Seyfarth and Cheney, 2015). Additionally, this type of habitat with dense vegetation and limited visibility, is believed to be one of the evolutionary forces that helped develop higher cognitive capacities in animal species such as great apes, as it required them to remember the spatiotemporal characteristics of feeding spots, while also impeding the sight of predators and other groups or individuals within the same species (Ban et al., 2019; Fichtel et al., 2025; Janmaat et al., 2021).

We predicted that if subjects took the presence of uninvolved bystanders into account, then their aggressive behavior should be different in the presence of socially important individuals in the audience. Among other categories, we classify as socially important individuals in regards of socially close individuals (kin-related or not) and higher-ranking individuals. For example, they should be especially prone to aggressive behavior in the presence of high-ranking observers, assuming that this is likely to secure future tolerance from them. On the other hand, they could be more aggressive toward others in the presence of friends (socially close) that might help them to reinforce their higher-ranking over others lower ranking than them. A key factor here is ‘calling’, because it could function to attract the attention of others, simply to gain reputation or to get potential supporters thus serving as a measure of intention. The more an individual would call during an aggression, the more likely is this aggression to be severe (chase or physically attack others), vs. mild (growling and staring at others). Similarly, the effect of the audience could be different in all the previous contexts depending on the severity of the aggression.

## Methods

### Study site and subjects

The study was conducted in Taï National Park in south-western Ivory Coast (5°50’N, 7°21’W). The park is the largest protected block of primary forest in West Africa and covers approximately 454,000 ha of continuous forest. The forest is classified as ‘tropical moist’ (Whitmore, 1990), with a mean annual temperature of 24 °C, a mean annual rainfall of 1,875 mm (average of 2012–2015; Taï Monkey Project long-term data) and a distinct dry season in December–January. The study area of about 7 km^2^ was situated near the western border of the park, approximately 20 km southeast of the township Taï.

Sooty mangabeys are mainly terrestrial and live in groups of up to 100 individuals, with large group spread and inter-individual distances. One consequence of this social system is that individuals only interact with a small proportion of the group at any given time. Although mangabey groups do not fission, individuals spend much of their time foraging in small parties going through the forest leaf-litter in search of food, such as insects or fallen Anthonota, Saccoglotis and Dialium fruits (Janmaat et al., 2006; McGraw et al., 2011; Range and Noë, 2002). Conflicts can occur in and outside of food patches, during which individuals can produce grunts, twitters, growls and screams (Quintero et al., 2022a; Range and Fischer, 2004). Prior studies on sooty mangabey aggression were mainly conducted in captivity, involving the introduction of new group members and formation of new groups (Bernstein, 1971; Bernstein and Gordon, 1974), situations that are unsuitable to test evolutionary questions about the function of aggression.

### Observational data

The study group’s home range contained a 2 km^2^ core area where groups of several monkey species had been studied since 1991 as part of a long-term research project (McGraw and Zuberbühler, 2007). The study group has been under constant observation since 1997 and is well habituated to human observers (Quintero et al., 2022a, 2022b; Range and Noë, 2002). Data collection was by following individuals from dawn to dusk over a period of 20 months (*N* = 92 observation days) from August 2013 to July 2014 and January to September 2015. During the study period the group size was around 80 individuals. Data collection was in the form of focal animal and instantaneous sampling (Altmann, 1974) on *N* = 33 adult individuals (*N* = 5 males; *N* = 28 females). We only worked with adults to avoid confounds due to ontogeny. Subjects were identified by physical features, such as scars, body size and general appearance. Focal samples lasted 60 min and individuals were not sampled twice during the same day. A total of *N* = 371 h of focal sampling was carried out on all *N* = 33 individuals (11.24 h ± 4.05 h/individual; mean ± SD; Supplementary Table S1). The observation times for the different individuals excluded out-of-sight, low-visibility or bad weather conditions. We curtailed the data further to only include interactions that occurred in full visibility between unambiguously identifiable individuals.

We defined a social interaction as an instance during which a focal animal approached, or was approached, by another individual to <1 m distance (see Bernstein, 1971; Quintero et al., 2022a; Range and Noë, 2002). When this was the case, we assumed that any call produced was socially directed. Call type discrimination followed the classification scheme by Range and Fischer (2004). We categorized a social interaction as ‘agonistic’ if it contained at least one aggressive behavior, e.g., slapping, chasing, biting, staring or lunging (see Quintero et al., 2022a). In addition, we collected information on the general activity of the focal individual every 15 min. For each social interaction, we also determined the audience composition every 15 min (‘neighbors’), i.e., the identity (ID) of every individual visible within a radius of about 10 m of the focal animal. These general activity factors were collected at the moment 15 min have passed (instant sampling) and not as the audience composition during that time frame.

### Statistical analyses

We were interested in what explained a focal animal’s agonistic behavior, in particular how it was linked to audience composition. Most agonistic interactions in sooty mangabeys are mild but occasionally interactants escalate and a conflict becomes severe. In two separate models, we therefore distinguished between (1) overall aggression (‘**agonistic**’) with all aggressive encounters during focal follows as data points and (2) severe aggression only (‘**severe**’) with the corresponding subset of data. Severe aggression is defined as every aggressive encounter where the aggressor is actively chasing the victim with or without physical contact.

If an encounter led to aggression, we considered it for the subsequent analyses and treated the approaching individual as the ‘aggressor’ and the approached individual as the ‘victim’. As predictor variables we used (a) whether the aggressor produced a call during the approach (binary, ‘**aggressor call**’), (b) whether the approached individual produced a call (binary, ‘**victim call**’), (c) the social status of the aggressor (Elo-rating score; numeric, ‘**ranking**’, Supplementary Table S1; see Neumann et al., 2011), (d) the size of the audience (numeric, ‘**neighbors**’), (e) the presence of higher-ranking individuals in the audience (binary, ‘**HR**’; defined by a neighbor’s Elo-rating score above the subject’s own score, Neumann et al., 2011), (f) the presence of bond partners in the audience (binary ‘**friend**’, defined by a dyadic composite sociality index (DSI) score >1 and ranging from 0 to 15, which we calculated using the socio-positive behaviors ‘approach’, ‘inspection’, ‘presenting groom’, ‘contact’, ‘groom’, ‘handle infant’ and ‘hug’(Supplementary Table S2; see Silk et al., 2013), (g) whether aggression was mild (stare, growl) or severe (chase, contact) (binary, ‘**severe**’), (h) whether the aggressor and the victim had visual contact for more than 20 s before the interaction (binary, ‘**sight**’). Finally, we included observer ID as a fixed factor to control for possible observer differences in data collection (*N* = 2; binary, ‘**observer**’). We included the IDs of the focal and the encountered animal, as well as the date, as random factors.

### Model 1: overall aggression

We used generalized linear mixed models (GLMM) with a binomial error structure to test variation in the occurrence of aggressive interactions with the response variable ‘agonistic’ (see Supplementary Information). To avoid singularity fit issues, we reran the models within a Bayesian framework using Wishart priors. After confirming that the results were similar, we reported the results from the Bayesian GLMMs. We used R v4.0.3 (R Core Team, 2020) with the ‘lme4’ (Bates et al., 2015) and ‘blme’ (Chung et al., 2013) packages for all GLMMs. Also, for all the models we ran diagnostics with the ‘DHARMa’ package (Hartig, 2022) using the simulateResiduals() function, the variance inflation using the vif() function from the ‘car’ package (Fox and Weisberg, 2018), the normal distribution of the residuals using the qqnorm() function from the ‘ggplot2’ package (Wickham, 2016), the normality of the random effects using the qqmath() function from the ‘lattice’ package (Sarkar, 2008), the singularity in the random effects structure with the isSingular() function from the package ‘lme4’, the influence of the random effects levels on the fixed effects with the influence() function from the ‘influence.ME’ package (Nieuwenhuis et al., 2012). The first model was set up to determine under what circumstances agonistic interactions were likely to occur (compared to friendly or neutral interactions). In this model, we did not distinguish between severe and mild aggression, so the variable ‘severe’ was not considered. We included interactions between the aggressor and the victim calling with the two audience factors ‘friend’ and ‘HR’, as well as with ‘sight’, except the control variables (observer ID and random factors) and the two excluded aggression-related variables. We ran the model with all interactions and then deleted one-by-one all non-significant ones, starting with the least significant interaction until arriving at a final model with only significant interactions. We included random intercepts for focal subject ID (IDF), encountered subject ID (IDE) and date. We did not include random slopes for Elo-rating because we used only Elo-ratings at the end of the study period, i.e., the ranks did not change. We then built an ‘informed null model’, which only comprised the fixed term ‘observer’. The random structure was identical to the full model (Supplementary Appendix 3). We then compared these models with a likelihood ratio test (Dobson and Barnett, 2018). If the comparison between full and null models revealed a significant difference, we explored the full model with regards to the predictors of interest, i.e., those in the full but not in the null model.

### Model 2: severe aggression

In order to understand the role of the audience in cases of aggression, we used generalized linear mixed models (GLMM) with a binomial error structure. We used all interactions that qualified as agonistic, with the response variable ‘severe’ (binary; mild = 0, severe = 1). We used all the same functions described for the previous model. We also ran the model with the same interactions described above and then deleted one-by-one the non-significant ones until we were left with the final model. We tested the significance of this model with a likelihood ratio test between the full and a null model, as mentioned above.

### Ethical note

We adhered to non-invasive data collection by following and observing individuals habituated to human observers in their natural habitat. Research permission and ethical clearance was granted by the Ministère de la Recherche Scientifique et Technique de Côte d’Ivoire. The methods are in line with the Animal Behavior Society Guidelines for the Use of Animals in Research.

## Results

### Overall aggression

We followed *N* = 33 individuals with an average of 674.4 min (11.24 h) per focal animal over *N* = 88 observation days. Individuals had about 4.6 directly observed social interactions per hour (*N* = 1,722 encounters; *N* = 371 observation hours), with about 1.6 agonistic encounters per hour (*N* = 595). As mentioned, we only considered encounters where we could see the interaction clearly and unambiguously identify the individuals, which led to a reduced dataset of *N* = 29 focal individuals during *N* = 52 days of observations (*N* = 47 days with at least one agonistic interaction). During the 52 observation days, we scored *N* = 887 social interactions, with *N* = 359 (40.5%) scored as agonistic, with either severe (*N* = 179) or mild (*N* = 180) aggression. During the *N* = 359 agonistic interactions, subjects produced *N* = 141 vocalizations (*N* = 100 growls, *N* = 20 screams, *N* = 19 grunts, *N* = 1 twitter, N = 1 copulation call).

**Figure 1:**
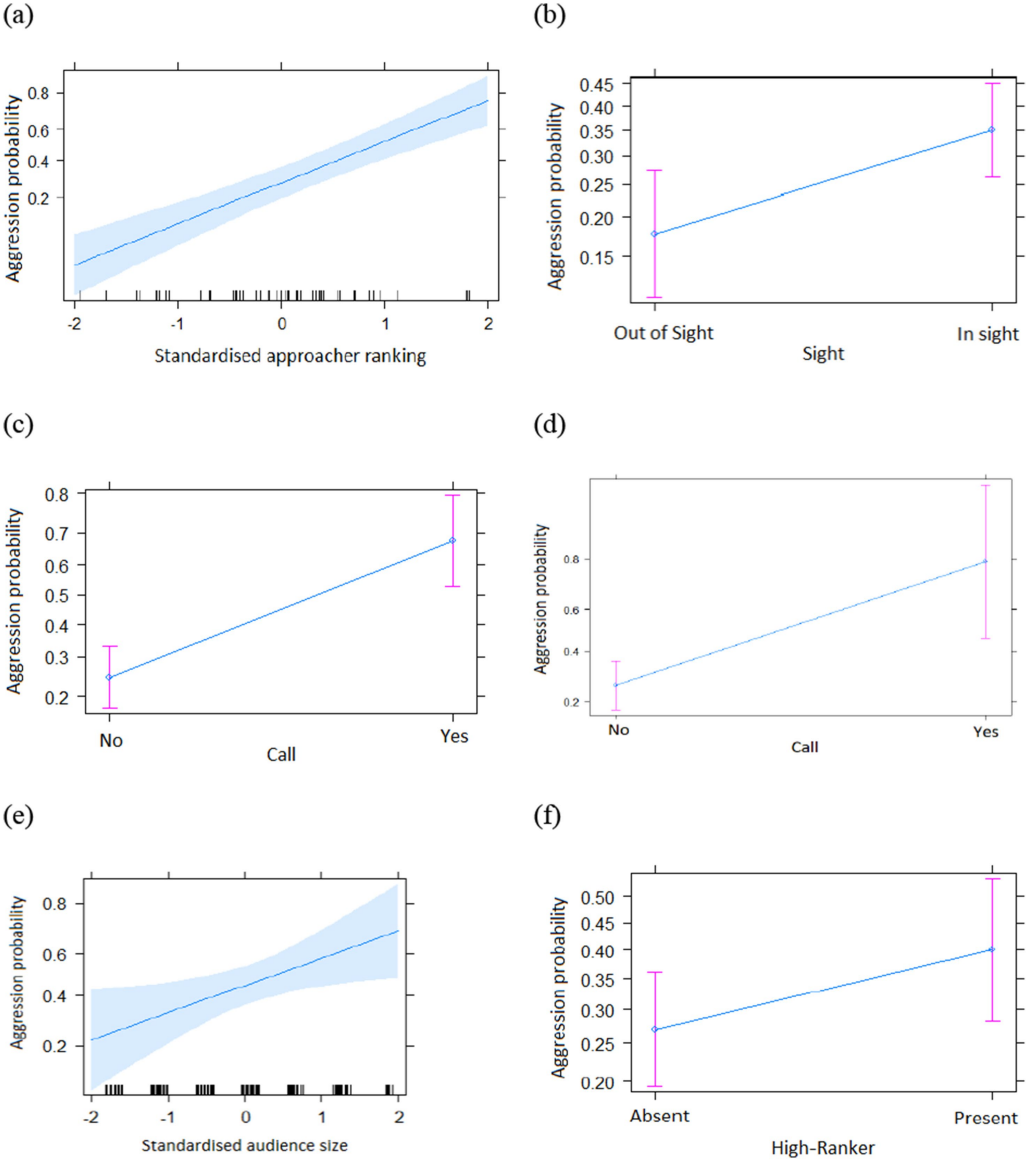
Overall probability of aggression as a function of different predictors (main effects only, **(a)** Ranking; **(b)** Sight; **(c)** Aggressor call; **(d)** Victim call; **(e)** Neighbours; **(f)** HR; **a–f**: means ± SE).

The full model with all the interactions was significantly different from the informed null model (χ^2^ = 211.67; df = 13, *p* < 0.001, see Supplementary Appendices 2, 3). We step-by-step removed the non-significant interactions which resulted in the final model with no interactions (Table 1). The final model was significantly different from the informed null model (χ^2^ = 203.1; df = 7, *p* < 0.001) (see Supplementary Table S3 for the 95% confidence intervals and Supplementary Table S4 for the VIF). We found that subjects were more likely to be aggressive during social encounters if they were (a) higher-ranking than the partner (Table 1 and Figure 1a), (b) in visual contact with the partner before the encounter (Table 1 and Figure 1b), (c) producing a call (Table 1 and Figure 1c), (d) encountering a victim that called (Table 1 and Figure 1d), (e) with a large audience (Table 1 and Figure 1e) and, crucially, (f) with a higher-ranking individual in the audience (Table 1 and Figure 1f). Finally, the presence of social allies had no significant influence on the overall aggression (Table 1).

**Table 1:** Model results for overall probability of aggression.

Variables	Estimate	SE	*Z*	Pr(>|z|)
Observer ID	0,01563	0,34,908	0,045	0,96,428
Ranking	1,08951	0,14,059	7,749	**9,24E-15**
Neighbors	0,32,866	0,10,244	3,208	**0,00133**
Aggressor call	1,90,724	0,2,823	6,756	**1,42E-11**
Friend	0,01626	0,26,605	0,061	0,95,125
Sight	1,01407	0,24,155	4,198	**2,69E-05**
Victim call	1,08131	0,35,336	3,06	**0,00221**
HR	0,70,744	0,23,148	3,056	**0,00224**

Values in bold are the results that are significant with at a 95% confidence level.

### Severe aggression

**Figure 2:**
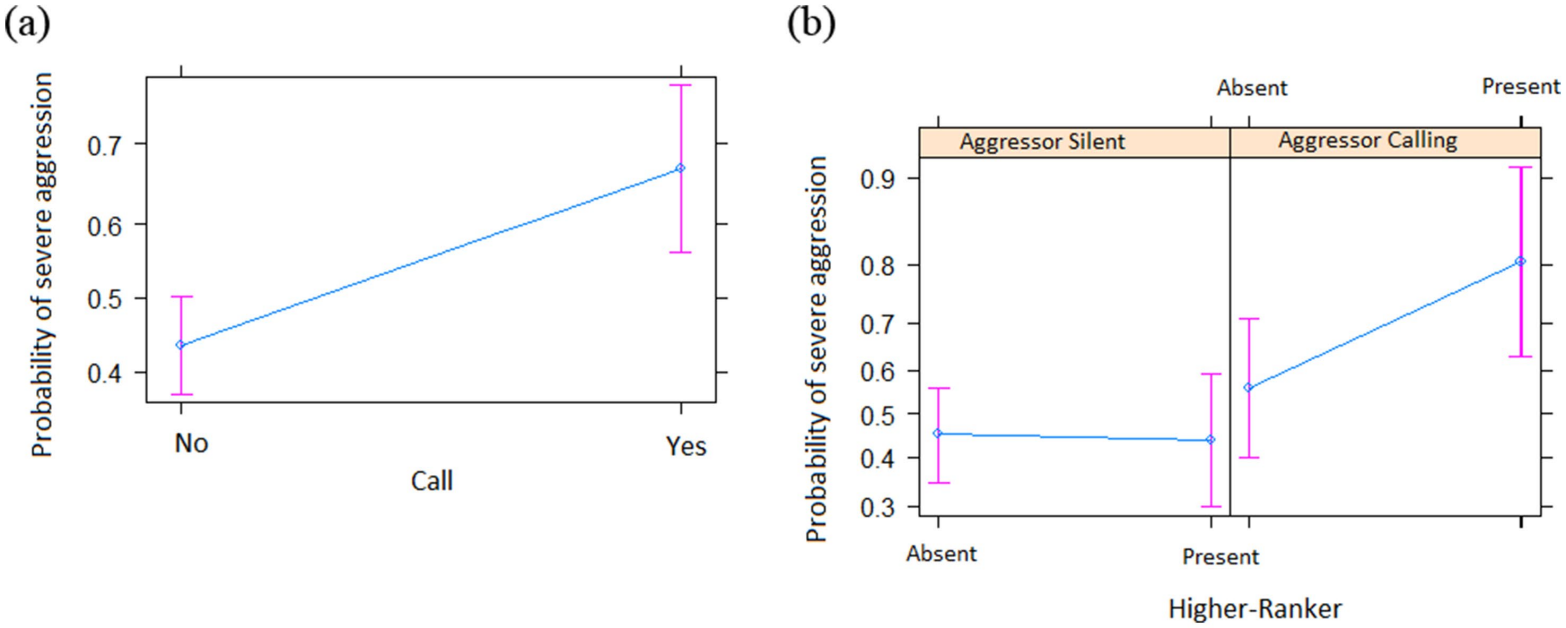
Probability of severe aggression as a function of different predictors (means ± SE). **(a)** Victim calling; **(b)** HR * Aggressor Calling.

*N* = 179 of *N* = 359 agonistic interactions that occurred between *N* = 29 individuals over *N* = 47 days qualified as severe. The full model was significantly different from the informed null model (χ^2^ = 35.46; df = 13, *p* < 0.001). As before, we step-by-step removed the non-significant interactions, which resulted in a final model with one interaction: aggressor calling * HR. The final model was significantly different from the informed null model (χ^2^ = 25.116; df = 8, *p* < 0.001) (see Supplementary Table S5 for the 95% confidence intervals and Supplementary Table S6 for the VIF). We found aggression was more likely to be severe when (a) the victim was calling (Table 2 and Figure 2a) and if the aggressor was calling and there was a higher-ranker in the audience (Table 2 and Figure 2b).

**Table 2:** Model results for the probability of severe aggression only.

**Variables**	**Estimate**	**SE**	***Z***	**Pr(>|z|)**
Observer ID	0,38,695	0,32,626	1,186	0,2,356
Ranking	0,16,106	0,15,529	1,037	0,2,997
Neighbors	0,22,914	0,13,185	1,738	0,0822
Friend	–0,03089	0,32,483	–0,095	0,9,242
Sight	–0,10,834	0,34,357	–0,315	0,7,525
Victim call	0,82,611	0,37,278	2,216	**0,0267**
HR	–0,05255	0,34,956	–0,15	0,8,805
Aggressor call	0,43,193	0,3,403	1,269	0,2043
HR: aggressor calling	1,22,992	0,60,663	2,027	**0,0426**

Values in bold are the results that are significant with at a 95% confidence level.

## Discussion

We were interested in sooty mangabey aggressive behavior to address a less explored problem in animal behavior, the degree to which aggressive interactions are determined by an observing audience. Aggression, whether if it is in a competition over resources (food or mating partners) or as a partner control mechanism, is typically examined as a dyadic process not taking into account the role of the audience on triggering these behaviors, especially in mild aggression. Additionally, these behaviors largely reserved to more dominant individuals in a group, the question is how middle and lower-ranking individuals achieve their goals. We hypothesized that one way of doing so was to display aggressive behavior in ways that uninvolved bystanders take note of the interaction.

To this end, we investigated the dynamics of aggressive behavior in sooty mangabeys in the presence of different audiences. As expected, and in line with the main function of aggression, we found that higher-ranking individuals were more aggressive than lower-ranking ones (probably to maintain social hierarchy), toward individuals in-sight within the last 15 s (individuals that are constantly on the radar, which would evidence certain intentionality rather than a simple emotional reaction), with a higher number of neighbors and that severe aggression was accompanied by vocal behavior suggests an element of planning and audience awareness (Bernstein, 1971; Janmaat et al., 2006; Mielke et al., 2017; Quintero et al., 2022a; Range et al., 2007; Range and Noë, 2002), as predicted by sociobiological theory (Clutton-Brock, 2016; Emlen and Oring, 1977). Crucially, we found effects that appeared to go beyond the predictions of dyadic resource conflicts and suggested that aggressors had an interest in being observed. We found that the presence of higher-ranking individuals in the audience was linked to agonistic interactions more generally (Figure 1f). The presence of a friend in the audience did not matter during overall or severe aggression and there was no interaction of this factor with any of the other investigated variables, suggesting that mangabeys attempt to ‘attract the attention’ of others by their ranking and not by their social bondness (Tables 1, 2). In conclusion, the patterns of aggression in sooty mangabeys found in this study complied with predictions of a basic function in resource competition but also with some form of social advertisement.

Audience effects appear to play a general role in primate decision-making. In a previous study (Quintero et al., 2022a), we found that the audience impacted on alarm calling, which was enhanced if socially important individuals were nearby, although this may be a group-specific or seasonal pattern (see Mielke et al., 2017). Audience effects are also very common in chimpanzees, such as when encountering snakes (Schel et al., 2013; Crockford et al., 2012), when discovering food (Slocombe et al., 2010) and during aggression (Slocombe and Zuberbühler, 2007). Relevant for the current study is that chimpanzees that are victims of aggression tend to exaggerate the nature of the attack, provided they are observed by high-ranking audiences (Slocombe et al., 2009), presumably to persuade nearby group members to intervene on behalf of them. This is similar to what we found, insofar as severe aggression was correlated with the aggressors calling when higher-rankers were in the audience (Table 2 and Figure 2b), which suggests an attempt to attract higher-rankers attention to the event, although the reasoning behind it remains unclear. Future studies will need to include what happens after these aggressive encounters, especially between the aggressor and the higher-rankers in the audience.

We also found that the sudden arrival of out-of-sight individuals did not increase the likelihood of aggression. Instead, individuals were more likely to be aggressive to those already in sight and higher-ranking group members watching, implying some sort of planning rather than impulsive reactions. This goes in line with the more strategic pattern seen in chimpanzees who actively try to draw attention from their audiences by modifying the acoustic structure of their calls (Slocombe and Zuberbühler, 2007).

Unfortunately, we were unable to study whether being aggressive in the presence of high-ranking bystanders leads to future tolerance from them, which is an argument that has also been made with regard for redirected aggression (Ito et al., 2018). Therefore, we cannot rule out the possibility that the presence of high-ranking individuals makes others more anxious and more likely to act aggressively.

Importantly, we did not find that the presence of friends in the audience had a measurable impact during aggressions (Tables 1, 2), which is similar to what has been reported before in the same species. Range and Noë (2002) found that coalitions in sooty mangabeys were rare (<4%) and mainly between high-ranking females against lower-ranking opponents, suggesting that victims cannot hope for support. Most coalitions only occurred once, providing further evidence against the idea that social bonds function to secure future support, as has been argued repeatedly for chimpanzees (Koyama et al., 2006; Watts, 2002) or vervet monkeys (Borgeaud and Bshary, 2015; Seyfarth and Cheney, 1984).

Our study suggests that mangabeys attempt to attract the attention of higher-rankers during ‘severe’ conflicts, but how does the audience play a role during aggressive behavior? As we did not measure immediate interactions between the higher-ranker and the approacher, we can only speculate. The cooperation literature has coined the notion of indirect (negative) reciprocity as another form of partner control to foster cooperation and future tolerance in others. If social interactions take place in front of others, then uninvolved bystanders will possibly remember the outcome for their future decision-making (Parrish et al., 2013). Having observed one individual being overly aggressive during food competition will likely result in this animal obtaining a reputation as being socially difficult, combative or even dangerous, which may increase the observer’s future tolerance toward this animal (Számadó et al., 2021). However, in sooty mangabeys’ society, higher-rankers in the audience would not need to be impressed by a ‘socially difficult’ individual attacking another so they can tolerate it later on during a competition over resources, because they are already in the ‘higher-rank’. In this case, it is possible that as it has been argued with redirected aggression (Watts et al., 2000), individuals are being aggressive toward others to divert the attention of a possible high-ranker aggressor, but before that ‘possible’ aggression toward them.

Humans evidently take into account the interaction history, the identity and social role of bystanders, which raises questions about the origins of such abilities in primate cognition (Zuberbühler, 2008). Recently, it has been argued that in order to handle such multidimensional problems primates follow more basic social scripts, which allow them to make accurate predictions about other group members’ future behaviors in most cases (Taylor et al., 2023). Yet, our data cannot distinguish between the main reputation hypotheses currently available. Nevertheless, the audience effects we observed may be due to some form of reputation building, and perhaps may even qualify as negative indirect reciprocity. Here, a key prediction from future research would be that, once an individual has performed an aggressive act in front of a higher-ranking bystander, it will gain future tolerance from the same individual, compared to cases when no aggressive acts were performed. Future analyses would therefore have to focus on the long-term effects of aggressive interactions, especially those that cannot be explained in terms of dyadic conflicts over access to resources.

To conclude, we have provided further evidence for a general primate propensity to adjust social behavior depending on the composition of the bystanding audience and react in the most appropriate way depending on the situation, which goes in line with recent works in this species (Quintero et al., 2022a). Primates arguably follow social scripts, which allows them to make predictions about the consequences of their own current behavior on future events (Taylor et al., 2023). The patterns described here are not in line with a notion of animal calls as hardwired or reflexive responses to specific stimuli, but appear to involve considerable amounts of social cognition, allowing individuals to make assessments of both ecological and social variables in ways that would meet criteria for intentionality.

## Data Availability

The datasets presented in this study can be found in online repositories. The names of the repository/repositories and accession number(s) can be found at: https://doi.org/10.6084/m9.figshare.20380347.
