# Supplementary material for: Audience effects in sooty mangabey agonistic behavior
Source: Front Psychol. 2025 Sep 30;16:1551210. doi: 10.3389/fpsyg.2025.1551210 (PMC12518070; doi:10.3389/fpsyg.2025.1551210)
Supplement: Supplementary file 1 [file Data_Sheet_1.docx]

**Supplementary Information**

**Full model with interactions for ‘Overall Aggression’**

Agonistic ~ Observer ID + ranking + neighbours + sight*friend + sight*HR + AC*friend + AC*HR + friend*VC + VC*HR + (1|IDF) + (1|IDE) + (1|Date)

**Final model for ‘Overall Aggression’**

Agonistic ~ Ranking + Neighbours + Sight + AC + Observer ID + Friend + VC + HR + (1|IDF) + (1|IDE) + (1|Date)

**Informed null model for ‘Overall Aggression’**

Agonistic ~ Observer ID + (1|IDF) + (1|IDE) + (1|Date)

**Full model with interactions for ‘Severe Aggression’**

Severe ~ Observer ID + Sight + AC + VC + Ranking + Friend + HR + Neighbours + sight*friend + sight*HR + AC*friend + AC*HR + friend*VC + VC*HR + (1|IDF) + (1|IDE) + (1|Date)

**Final model for ‘Severe Aggression’**

Severe ~ Sight + AC + VC + Ranking + Friend + HR + Neighbours + Observer ID + AC*HR + (1|IDF) + (1|IDE) + (1|Date)

**Informed null model for ‘Severe Aggression’**

Severe ~ Observer ID + (1|IDF) + (1|IDE) + (1|Date)

**Supplementary Table S1.** Observation time for the different focal animals including rank and sex

| **Name** | **Sex** | **Elo Score** | **Observation time (HH:MM)** |
| --- | --- | --- | --- |
| **Norm** | **Male** | **1542** | **15:10** |
| **Michael** | **Male** | **1495** | **10:16** |
| **Drogba** | **Male** | **1405** | **3:06** |
| **Appo** | **Male** | **1217** | **8:20** |
| **Falcao** | **Male** | **1127** | **5:52** |
| **Hilda** | **Female** | **1411** | **16:02** |
| **Agatha** | **Female** | **1254** | **10:39** |
| **Helena** | **Female** | **1204** | **11:01** |
| **Nina** | **Female** | **1194** | **13:36** |
| **Sophie** | **Female** | **1160** | **6:05** |
| **Libia** | **Female** | **1090** | **13:44** |
| **Dorothee** | **Female** | **1079** | **6:10** |
| **Odile** | **Female** | **1072** | **14:10** |
| **Lana** | **Female** | **1046** | **11:29** |
| **Ivette** | **Female** | **1022** | **7:23** |
| **Tina** | **Female** | **1014** | **11:50** |
| **Emma** | **Female** | **1003** | **9:56** |
| **Mary** | **Female** | **950** | **12:35** |
| **Fiji** | **Female** | **921** | **12:26** |
| **Lama** | **Female** | **908** | **10:02** |
| **Mona** | **Female** | **906** | **19:05** |
| **Oval** | **Female** | **900** | **16:07** |
| **Fiona** | **Female** | **850** | **14:30** |
| **Sri Lanka** | **Female** | **849** | **5:53** |
| **Elizabeth** | **Female** | **830** | **16:34** |
| **Teresa** | **Female** | **761** | **11:00** |
| **Svenia** | **Female** | **754** | **11:59** |
| **Jeanne** | **Female** | **741** | **10:21** |
| **Johanna** | **Female** | **735** | **13:43** |
| **Caterina** | **Female** | **733** | **4:02** |
| **Veronica** | **Female** | **690** | **8:37** |
| **Edith** | **Female** | **626** | **9:49** |
| **Tatiana** | **Female** | **568** | **19:22** |
| **Total** |  |  | **370:56** |

**Supplementary Table S2.** Dyadic composite sociality index (DSI) scores calculated from socio-positive behaviours ‘approach’, ‘inspection’, ‘presenting groom’, ‘contact’, ‘groom’, ‘handle infant’ and ‘hug’. In this table we only present dyads that had a DSI score higher than 0. RT’ are the rates for each behaviour. To calculate the DSI, the interaction rates for these behaviours were collected for each dyad, and then these rates were combined to form the final DSI score, which indicates the overall strength of the relationship.

| **i1** | **i2** | **approach** | **buttinsp** | **buttpres** | **contact** | **groom** | **handle_baby** | **hug** | **approach.rt** | **buttinsp.rt** | **buttpres.rt** | **contact.rt** | **groom.rt** | **handle_baby.rt** | **hug.rt** | **DSI** | **zDSI** |
| --- | --- | --- | --- | --- | --- | --- | --- | --- | --- | --- | --- | --- | --- | --- | --- | --- | --- |
| Agatha | Fiona | 4 | 0 | 0 | 0 | 273 | 0 | 0 | 14,29 | 0 | 0 | 0 | 38,35 | 0 | 0 | 7,52 | 1,69 |
| Agatha | Hilda | 1 | 0 | 0 | 0 | 519 | 0 | 0 | 3,57 | 0 | 0 | 0 | 72,90 | 0 | 0 | 10,92 | 1,83 |
| Agatha | Johanna | 1 | 0 | 0 | 0 | 17 | 0 | 0 | 3,57 | 0 | 0 | 0 | 2,39 | 0 | 0 | 0,85 | 0,07 |
| Agatha | Lana | 1 | 0 | 0 | 1 | 0 | 0 | 0 | 3,57 | 0 | 0 | 10,58 | 0 | 0 | 0 | 2,02 | 0,47 |
| Agatha | Odile | 1 | 0 | 0 | 0 | 0 | 0 | 1 | 3,57 | 0 | 0 | 0 | 0 | 0 | 23,38 | 3,85 | 0,66 |
| Agatha | Veronica | 1 | 0 | 0 | 0 | 0 | 1 | 0 | 3,57 | 0 | 0 | 0 | 0 | 80,14 | 0 | 11,96 | 1,30 |
| Appo | Mona | 1 | 1 | 0 | 0 | 0 | 0 | 0 | 3,57 | 43,15 | 0 | 0 | 0 | 0 | 0 | 6,68 | 0,96 |
| Appo | Nina | 1 | 1 | 0 | 0 | 0 | 0 | 0 | 3,57 | 43,15 | 0 | 0 | 0 | 0 | 0 | 6,68 | 0,96 |
| Appo | Odile | 1 | 0 | 1 | 0 | 0 | 0 | 0 | 3,57 | 0 | 24,39 | 0 | 0 | 0 | 0 | 3,99 | 0,73 |
| Caterina | Edith | 2 | 0 | 0 | 0 | 284 | 0 | 1 | 7,15 | 0 | 0 | 0 | 39,89 | 0 | 23,38 | 10,06 | 1,90 |
| Caterina | Elizabeth | 1 | 0 | 0 | 0 | 0 | 0 | 1 | 3,57 | 0 | 0 | 0 | 0 | 0 | 23,38 | 3,85 | 0,66 |
| Caterina | Falcao | 0 | 0 | 1 | 0 | 0 | 0 | 0 | 0 | 0 | 24,39 | 0 | 0 | 0 | 0 | 3,48 | 0,49 |
| Caterina | Hilda | 1 | 1 | 0 | 0 | 0 | 0 | 0 | 3,57 | 43,15 | 0 | 0 | 0 | 0 | 0 | 6,68 | 0,96 |
| Caterina | Lana | 2 | 0 | 0 | 0 | 10 | 0 | 1 | 7,15 | 0 | 0 | 0 | 1,40 | 0 | 23,38 | 4,56 | 0,94 |
| Caterina | Mary | 2 | 0 | 0 | 0 | 77 | 0 | 1 | 7,15 | 0 | 0 | 0 | 10,82 | 0 | 23,38 | 5,91 | 1,17 |
| Caterina | Mona | 2 | 0 | 0 | 0 | 0 | 0 | 2 | 7,15 | 0 | 0 | 0 | 0 | 0 | 46,75 | 7,70 | 1,55 |
| Caterina | Odile | 1 | 0 | 0 | 1 | 0 | 0 | 0 | 3,57 | 0 | 0 | 10,58 | 0 | 0 | 0 | 2,02 | 0,47 |
| Caterina | Sophie | 2 | 0 | 0 | 0 | 25 | 0 | 1 | 7,15 | 0 | 0 | 0 | 3,51 | 0 | 23,38 | 4,86 | 0,99 |
| Caterina | Svenia | 2 | 0 | 0 | 1 | 0 | 0 | 1 | 7,15 | 0 | 0 | 10,58 | 0 | 0 | 23,38 | 5,87 | 1,36 |
| Caterina | Tatiana | 1 | 1 | 0 | 0 | 0 | 0 | 0 | 3,57 | 43,15 | 0 | 0 | 0 | 0 | 0 | 6,68 | 0,96 |
| Dorothee | Hilda | 1 | 0 | 0 | 1 | 0 | 0 | 0 | 3,57 | 0 | 0 | 10,58 | 0 | 0 | 0 | 2,02 | 0,47 |
| Dorothee | Lama | 1 | 0 | 0 | 0 | 0 | 1 | 0 | 3,57 | 0 | 0 | 0 | 0 | 80,14 | 0 | 11,96 | 1,30 |
| Drogba | Emma | 1 | 0 | 1 | 0 | 0 | 0 | 0 | 3,57 | 0 | 24,39 | 0 | 0 | 0 | 0 | 3,99 | 0,73 |
| Drogba | Hilda | 1 | 0 | 1 | 0 | 0 | 0 | 0 | 3,57 | 0 | 24,39 | 0 | 0 | 0 | 0 | 3,99 | 0,73 |
| Drogba | Libia | 1 | 1 | 0 | 0 | 0 | 0 | 0 | 3,57 | 43,15 | 0 | 0 | 0 | 0 | 0 | 6,68 | 0,96 |
| Drogba | Oval | 1 | 1 | 0 | 0 | 0 | 0 | 0 | 3,57 | 43,15 | 0 | 0 | 0 | 0 | 0 | 6,68 | 0,96 |
| Edith | Falcao | 0 | 0 | 1 | 0 | 0 | 0 | 0 | 0 | 0 | 24,39 | 0 | 0 | 0 | 0 | 3,48 | 0,49 |
| Edith | Fiji | 1 | 0 | 1 | 0 | 0 | 0 | 0 | 3,57 | 0 | 24,39 | 0 | 0 | 0 | 0 | 3,99 | 0,73 |
| Edith | Fiona | 1 | 0 | 0 | 0 | 0 | 0 | 1 | 3,57 | 0 | 0 | 0 | 0 | 0 | 23,38 | 3,85 | 0,66 |
| Edith | Helena | 1 | 0 | 0 | 0 | 0 | 0 | 1 | 3,57 | 0 | 0 | 0 | 0 | 0 | 23,38 | 3,85 | 0,66 |
| Edith | Jeanne | 1 | 0 | 0 | 1 | 0 | 0 | 0 | 3,57 | 0 | 0 | 10,58 | 0 | 0 | 0 | 2,02 | 0,47 |
| Edith | Johanna | 2 | 0 | 1 | 0 | 49 | 0 | 0 | 7,15 | 0 | 24,39 | 0 | 6,88 | 0 | 0 | 5,49 | 1,14 |
| Edith | Lama | 1 | 0 | 1 | 0 | 0 | 0 | 0 | 3,57 | 0 | 24,39 | 0 | 0 | 0 | 0 | 3,99 | 0,73 |
| Edith | Libia | 1 | 0 | 0 | 1 | 0 | 0 | 0 | 3,57 | 0 | 0 | 10,58 | 0 | 0 | 0 | 2,02 | 0,47 |
| Edith | Mona | 3 | 0 | 0 | 2 | 0 | 0 | 1 | 10,72 | 0 | 0 | 21,17 | 0 | 0 | 23,38 | 7,89 | 2,06 |
| Edith | Sophie | 1 | 0 | 0 | 0 | 55 | 0 | 0 | 3,57 | 0 | 0 | 0 | 7,73 | 0 | 0 | 1,61 | 0,20 |
| Edith | Svenia | 1 | 0 | 0 | 0 | 0 | 0 | 1 | 3,57 | 0 | 0 | 0 | 0 | 0 | 23,38 | 3,85 | 0,66 |
| Edith | Tatiana | 1 | 0 | 0 | 1 | 0 | 0 | 0 | 3,57 | 0 | 0 | 10,58 | 0 | 0 | 0 | 2,02 | 0,47 |
| Edith | Teresa | 1 | 0 | 1 | 0 | 0 | 0 | 0 | 3,57 | 0 | 24,39 | 0 | 0 | 0 | 0 | 3,99 | 0,73 |
| Elizabeth | Falcao | 0 | 0 | 0 | 0 | 103 | 0 | 0 | 0 | 0 | 0 | 0 | 14,47 | 0 | 0 | 2,07 | 0,13 |
| Elizabeth | Jeanne | 1 | 0 | 0 | 1 | 0 | 0 | 0 | 3,57 | 0 | 0 | 10,58 | 0 | 0 | 0 | 2,02 | 0,47 |
| Elizabeth | Lana | 1 | 0 | 1 | 0 | 0 | 0 | 0 | 3,57 | 0 | 24,39 | 0 | 0 | 0 | 0 | 3,99 | 0,73 |
| Elizabeth | Tatiana | 2 | 0 | 0 | 0 | 16 | 0 | 0 | 7,15 | 0 | 0 | 0 | 2,25 | 0 | 0 | 1,34 | 0,31 |
| Elizabeth | Teresa | 1 | 0 | 0 | 1 | 0 | 0 | 0 | 3,57 | 0 | 0 | 10,58 | 0 | 0 | 0 | 2,02 | 0,47 |
| Emma | Fiji | 1 | 0 | 0 | 0 | 0 | 0 | 1 | 3,57 | 0 | 0 | 0 | 0 | 0 | 23,38 | 3,85 | 0,66 |
| Emma | Fiona | 1 | 0 | 0 | 0 | 0 | 1 | 0 | 3,57 | 0 | 0 | 0 | 0 | 80,14 | 0 | 11,96 | 1,30 |
| Emma | Johanna | 1 | 0 | 0 | 1 | 0 | 0 | 0 | 3,57 | 0 | 0 | 10,58 | 0 | 0 | 0 | 2,02 | 0,47 |
| Emma | Lama | 1 | 0 | 0 | 0 | 75 | 0 | 0 | 3,57 | 0 | 0 | 0 | 10,53 | 0 | 0 | 2,02 | 0,27 |
| Emma | Libia | 2 | 0 | 0 | 1 | 161 | 0 | 0 | 7,15 | 0 | 0 | 10,58 | 22,61 | 0 | 0 | 5,76 | 1,28 |
| Emma | Mary | 1 | 0 | 0 | 1 | 0 | 0 | 0 | 3,57 | 0 | 0 | 10,58 | 0 | 0 | 0 | 2,02 | 0,47 |
| Emma | Nina | 1 | 0 | 0 | 0 | 249 | 0 | 0 | 3,57 | 0 | 0 | 0 | 34,97 | 0 | 0 | 5,51 | 0,88 |
| Emma | Odile | 1 | 0 | 0 | 1 | 0 | 0 | 0 | 3,57 | 0 | 0 | 10,58 | 0 | 0 | 0 | 2,02 | 0,47 |
| Falcao | Fiji | 0 | 1 | 0 | 0 | 74 | 0 | 0 | 0 | 43,15 | 0 | 0 | 10,39 | 0 | 0 | 7,65 | 0,98 |
| Falcao | Hilda | 0 | 0 | 1 | 0 | 0 | 0 | 0 | 0 | 0 | 24,39 | 0 | 0 | 0 | 0 | 3,48 | 0,49 |
| Falcao | Tina | 0 | 0 | 0 | 0 | 165 | 0 | 0 | 0 | 0 | 0 | 0 | 23,18 | 0 | 0 | 3,31 | 0,35 |
| Fiji | Fiona | 1 | 0 | 0 | 0 | 11 | 0 | 0 | 3,57 | 0 | 0 | 0 | 1,55 | 0 | 0 | 0,73 | 0,05 |
| Fiji | Johanna | 1 | 0 | 1 | 0 | 0 | 0 | 0 | 3,57 | 0 | 24,39 | 0 | 0 | 0 | 0 | 3,99 | 0,73 |
| Fiji | Lana | 2 | 0 | 0 | 0 | 0 | 0 | 2 | 7,15 | 0 | 0 | 0 | 0 | 0 | 46,75 | 7,70 | 1,55 |
| Fiji | Libia | 1 | 0 | 0 | 0 | 9 | 0 | 0 | 3,57 | 0 | 0 | 0 | 1,26 | 0 | 0 | 0,69 | 0,04 |
| Fiji | Mary | 1 | 0 | 0 | 1 | 0 | 0 | 0 | 3,57 | 0 | 0 | 10,58 | 0 | 0 | 0 | 2,02 | 0,47 |
| Fiji | Odile | 1 | 0 | 0 | 1 | 0 | 0 | 0 | 3,57 | 0 | 0 | 10,58 | 0 | 0 | 0 | 2,02 | 0,47 |
| Fiji | Oval | 1 | 0 | 0 | 1 | 0 | 0 | 0 | 3,57 | 0 | 0 | 10,58 | 0 | 0 | 0 | 2,02 | 0,47 |
| Fiji | Tatiana | 1 | 0 | 0 | 1 | 0 | 0 | 0 | 3,57 | 0 | 0 | 10,58 | 0 | 0 | 0 | 2,02 | 0,47 |
| Fiona | Helena | 1 | 0 | 0 | 0 | 281 | 0 | 0 | 3,57 | 0 | 0 | 0 | 39,47 | 0 | 0 | 6,15 | 1,00 |
| Fiona | Hilda | 1 | 0 | 0 | 0 | 0 | 0 | 1 | 3,57 | 0 | 0 | 0 | 0 | 0 | 23,38 | 3,85 | 0,66 |
| Fiona | Lama | 1 | 0 | 0 | 1 | 0 | 0 | 0 | 3,57 | 0 | 0 | 10,58 | 0 | 0 | 0 | 2,02 | 0,47 |
| Fiona | Lana | 1 | 0 | 1 | 0 | 0 | 0 | 0 | 3,57 | 0 | 24,39 | 0 | 0 | 0 | 0 | 3,99 | 0,73 |
| Fiona | Libia | 1 | 0 | 0 | 1 | 0 | 0 | 0 | 3,57 | 0 | 0 | 10,58 | 0 | 0 | 0 | 2,02 | 0,47 |
| Fiona | Michael | 2 | 0 | 0 | 0 | 13 | 0 | 0 | 7,15 | 0 | 0 | 0 | 1,83 | 0 | 0 | 1,28 | 0,30 |
| Fiona | Tatiana | 1 | 0 | 0 | 0 | 5 | 0 | 0 | 3,57 | 0 | 0 | 0 | 0,70 | 0 | 0 | 0,61 | 0,03 |
| Fiona | Tina | 1 | 0 | 0 | 1 | 0 | 0 | 0 | 3,57 | 0 | 0 | 10,58 | 0 | 0 | 0 | 2,02 | 0,47 |
| Fiona | Veronica | 1 | 0 | 0 | 1 | 0 | 0 | 0 | 3,57 | 0 | 0 | 10,58 | 0 | 0 | 0 | 2,02 | 0,47 |
| Helena | Lama | 1 | 0 | 0 | 1 | 0 | 0 | 0 | 3,57 | 0 | 0 | 10,58 | 0 | 0 | 0 | 2,02 | 0,47 |
| Helena | Libia | 1 | 0 | 0 | 1 | 0 | 0 | 0 | 3,57 | 0 | 0 | 10,58 | 0 | 0 | 0 | 2,02 | 0,47 |
| Helena | Nina | 3 | 0 | 1 | 1 | 0 | 0 | 1 | 10,72 | 0 | 24,39 | 10,58 | 0 | 0 | 23,38 | 9,87 | 2,32 |
| Helena | Sophie | 1 | 0 | 0 | 0 | 112 | 0 | 0 | 3,57 | 0 | 0 | 0 | 15,73 | 0 | 0 | 2,76 | 0,40 |
| Helena | Svenia | 1 | 0 | 0 | 1 | 0 | 0 | 0 | 3,57 | 0 | 0 | 10,58 | 0 | 0 | 0 | 2,02 | 0,47 |
| Helena | Tatiana | 1 | 0 | 0 | 0 | 0 | 0 | 1 | 3,57 | 0 | 0 | 0 | 0 | 0 | 23,38 | 3,85 | 0,66 |
| Helena | Teresa | 1 | 0 | 1 | 0 | 0 | 0 | 0 | 3,57 | 0 | 24,39 | 0 | 0 | 0 | 0 | 3,99 | 0,73 |
| Hilda | Mary | 1 | 0 | 0 | 0 | 160 | 0 | 0 | 3,57 | 0 | 0 | 0 | 22,47 | 0 | 0 | 3,72 | 0,57 |
| Hilda | Nina | 2 | 0 | 0 | 1 | 14 | 0 | 0 | 7,15 | 0 | 0 | 10,58 | 1,97 | 0 | 0 | 2,81 | 0,76 |
| Hilda | Norm | 2 | 0 | 0 | 1 | 26 | 0 | 0 | 7,15 | 0 | 0 | 10,58 | 3,65 | 0 | 0 | 3,05 | 0,80 |
| Hilda | Sophie | 2 | 0 | 0 | 0 | 52 | 0 | 0 | 7,15 | 0 | 0 | 0 | 7,30 | 0 | 0 | 2,06 | 0,43 |
| Hilda | Tatiana | 1 | 0 | 0 | 1 | 0 | 0 | 0 | 3,57 | 0 | 0 | 10,58 | 0 | 0 | 0 | 2,02 | 0,47 |
| Ivette | Teresa | 1 | 0 | 1 | 0 | 0 | 0 | 0 | 3,57 | 0 | 24,39 | 0 | 0 | 0 | 0 | 3,99 | 0,73 |
| Jeanne | Johanna | 1 | 0 | 0 | 1 | 0 | 0 | 0 | 3,57 | 0 | 0 | 10,58 | 0 | 0 | 0 | 2,02 | 0,47 |
| Jeanne | Michael | 1 | 0 | 0 | 0 | 17 | 0 | 0 | 3,57 | 0 | 0 | 0 | 2,39 | 0 | 0 | 0,85 | 0,07 |
| Jeanne | Sri Lanka | 1 | 0 | 0 | 1 | 0 | 0 | 0 | 3,57 | 0 | 0 | 10,58 | 0 | 0 | 0 | 2,02 | 0,47 |
| Jeanne | Tatiana | 1 | 0 | 0 | 1 | 0 | 0 | 0 | 3,57 | 0 | 0 | 10,58 | 0 | 0 | 0 | 2,02 | 0,47 |
| Johanna | Lama | 1 | 0 | 1 | 0 | 0 | 0 | 0 | 3,57 | 0 | 24,39 | 0 | 0 | 0 | 0 | 3,99 | 0,73 |
| Johanna | Lana | 1 | 0 | 0 | 0 | 0 | 0 | 1 | 3,57 | 0 | 0 | 0 | 0 | 0 | 23,38 | 3,85 | 0,66 |
| Johanna | Mary | 1 | 0 | 0 | 1 | 0 | 0 | 0 | 3,57 | 0 | 0 | 10,58 | 0 | 0 | 0 | 2,02 | 0,47 |
| Johanna | Odile | 1 | 0 | 0 | 1 | 0 | 0 | 0 | 3,57 | 0 | 0 | 10,58 | 0 | 0 | 0 | 2,02 | 0,47 |
| Johanna | Tatiana | 3 | 0 | 0 | 1 | 68 | 0 | 0 | 10,72 | 0 | 0 | 10,58 | 9,55 | 0 | 0 | 4,41 | 1,19 |
| Johanna | Teresa | 1 | 0 | 0 | 0 | 0 | 0 | 1 | 3,57 | 0 | 0 | 0 | 0 | 0 | 23,38 | 3,85 | 0,66 |
| Lama | Lana | 2 | 0 | 1 | 0 | 0 | 1 | 0 | 7,15 | 0 | 24,39 | 0 | 0 | 80,14 | 0 | 15,95 | 2,26 |
| Lama | Libia | 2 | 0 | 0 | 1 | 143 | 0 | 0 | 7,15 | 0 | 0 | 10,58 | 20,09 | 0 | 0 | 5,40 | 1,21 |
| Lama | Mona | 1 | 0 | 0 | 1 | 0 | 0 | 0 | 3,57 | 0 | 0 | 10,58 | 0 | 0 | 0 | 2,02 | 0,47 |
| Lama | Nina | 3 | 0 | 0 | 1 | 299 | 0 | 0 | 10,72 | 0 | 0 | 10,58 | 42,00 | 0 | 0 | 9,04 | 2,00 |
| Lama | Oval | 1 | 0 | 0 | 1 | 0 | 0 | 0 | 3,57 | 0 | 0 | 10,58 | 0 | 0 | 0 | 2,02 | 0,47 |
| Lama | Tatiana | 1 | 1 | 0 | 0 | 0 | 0 | 0 | 3,57 | 43,15 | 0 | 0 | 0 | 0 | 0 | 6,68 | 0,96 |
| Lama | Tina | 1 | 0 | 0 | 1 | 0 | 0 | 0 | 3,57 | 0 | 0 | 10,58 | 0 | 0 | 0 | 2,02 | 0,47 |
| Lana | Libia | 2 | 0 | 1 | 1 | 0 | 0 | 0 | 7,15 | 0 | 24,39 | 10,58 | 0 | 0 | 0 | 6,02 | 1,43 |
| Lana | Mary | 2 | 0 | 0 | 0 | 18 | 0 | 1 | 7,15 | 0 | 0 | 0 | 2,53 | 0 | 23,38 | 4,72 | 0,96 |
| Lana | Michael | 1 | 1 | 0 | 0 | 0 | 0 | 0 | 3,57 | 43,15 | 0 | 0 | 0 | 0 | 0 | 6,68 | 0,96 |
| Lana | Mona | 1 | 0 | 0 | 0 | 195 | 0 | 0 | 3,57 | 0 | 0 | 0 | 27,39 | 0 | 0 | 4,42 | 0,69 |
| Lana | Norm | 1 | 0 | 1 | 0 | 0 | 0 | 0 | 3,57 | 0 | 24,39 | 0 | 0 | 0 | 0 | 3,99 | 0,73 |
| Libia | Mary | 2 | 0 | 0 | 0 | 0 | 1 | 1 | 7,15 | 0 | 0 | 0 | 0 | 80,14 | 23,38 | 15,81 | 2,19 |
| Libia | Odile | 1 | 0 | 0 | 0 | 316 | 0 | 0 | 3,57 | 0 | 0 | 0 | 44,39 | 0 | 0 | 6,85 | 1,12 |
| Libia | Svenia | 1 | 0 | 0 | 1 | 0 | 0 | 0 | 3,57 | 0 | 0 | 10,58 | 0 | 0 | 0 | 2,02 | 0,47 |
| Libia | Tatiana | 1 | 0 | 0 | 1 | 0 | 0 | 0 | 3,57 | 0 | 0 | 10,58 | 0 | 0 | 0 | 2,02 | 0,47 |
| Libia | Veronica | 1 | 0 | 0 | 1 | 0 | 0 | 0 | 3,57 | 0 | 0 | 10,58 | 0 | 0 | 0 | 2,02 | 0,47 |
| Mary | Michael | 2 | 0 | 0 | 0 | 83 | 0 | 0 | 7,15 | 0 | 0 | 0 | 11,66 | 0 | 0 | 2,69 | 0,54 |
| Mary | Nina | 1 | 0 | 0 | 0 | 0 | 0 | 1 | 3,57 | 0 | 0 | 0 | 0 | 0 | 23,38 | 3,85 | 0,66 |
| Mary | Odile | 1 | 0 | 0 | 1 | 0 | 0 | 0 | 3,57 | 0 | 0 | 10,58 | 0 | 0 | 0 | 2,02 | 0,47 |
| Mary | Tatiana | 1 | 0 | 1 | 0 | 0 | 0 | 0 | 3,57 | 0 | 24,39 | 0 | 0 | 0 | 0 | 3,99 | 0,73 |
| Mary | Teresa | 1 | 0 | 1 | 0 | 0 | 0 | 0 | 3,57 | 0 | 24,39 | 0 | 0 | 0 | 0 | 3,99 | 0,73 |
| Michael | Nina | 1 | 1 | 0 | 0 | 0 | 0 | 0 | 3,57 | 43,15 | 0 | 0 | 0 | 0 | 0 | 6,68 | 0,96 |
| Michael | Svenia | 1 | 1 | 0 | 0 | 0 | 0 | 0 | 3,57 | 43,15 | 0 | 0 | 0 | 0 | 0 | 6,68 | 0,96 |
| Mona | Svenia | 1 | 1 | 0 | 0 | 0 | 0 | 0 | 3,57 | 43,15 | 0 | 0 | 0 | 0 | 0 | 6,68 | 0,96 |
| Mona | Teresa | 1 | 0 | 0 | 1 | 0 | 0 | 0 | 3,57 | 0 | 0 | 10,58 | 0 | 0 | 0 | 2,02 | 0,47 |
| Nina | Sophie | 1 | 0 | 0 | 1 | 0 | 0 | 0 | 3,57 | 0 | 0 | 10,58 | 0 | 0 | 0 | 2,02 | 0,47 |
| Nina | Tina | 1 | 0 | 0 | 0 | 0 | 1 | 0 | 3,57 | 0 | 0 | 0 | 0 | 80,14 | 0 | 11,96 | 1,30 |
| Nina | Veronica | 1 | 0 | 0 | 0 | 0 | 1 | 0 | 3,57 | 0 | 0 | 0 | 0 | 80,14 | 0 | 11,96 | 1,30 |
| Odile | Solange | 1 | 0 | 1 | 0 | 0 | 0 | 0 | 3,57 | 0 | 24,39 | 0 | 0 | 0 | 0 | 3,99 | 0,73 |
| Odile | Veronica | 1 | 0 | 0 | 1 | 0 | 0 | 0 | 3,57 | 0 | 0 | 10,58 | 0 | 0 | 0 | 2,02 | 0,47 |
| Svenia | Tatiana | 3 | 1 | 0 | 2 | 0 | 0 | 0 | 10,72 | 43,15 | 0 | 21,17 | 0 | 0 | 0 | 10,72 | 2,36 |
| Svenia | Teresa | 1 | 0 | 0 | 0 | 20 | 0 | 0 | 3,57 | 0 | 0 | 0 | 2,81 | 0 | 0 | 0,91 | 0,08 |
| Tatiana | Teresa | 2 | 0 | 0 | 2 | 0 | 0 | 0 | 7,15 | 0 | 0 | 21,17 | 0 | 0 | 0 | 4,05 | 1,17 |

**Supplementary Table S3. 95% Confidence Intervals for the ‘Overall aggression’ final model (Significant values in bold)**

| **Term** | **2.5 %** | **97.5 %** |
| --- | --- | --- |
| (Intercept) | -2,73749 | -1,42123 |
| Observer ID | -0,59093 | 0,750349 |
| **Ranking** | **0,81387** | **1,314372** |
| **Neighbours** | **0,141565** | **0,548638** |
| **Aggressor Call** | **1,349905** | **2,491452** |
| Friend | -0,52653 | 0,515412 |
| **Sight** | **0,520667** | **1,542122** |
| **Victim Call** | **0,465275** | **1,822395** |
| **HR** | **0,214735** | **1,158823** |

**Supplementary Table S4. Variance Inflation Factors for each predictor from the ‘Overall aggression’ final model**

| **Observer ID** | **Ranking** | **Neighbours** | **Sight** | **AC** | **Friend** | **VC** | **HR** |
| --- | --- | --- | --- | --- | --- | --- | --- |
| 1.280106 | 1.120159 | 1.070862 | 1.239880 | 1.066717 | 1.072550 | 1.051853 | 1.201837 |

**Supplementary Table S5. 95% Confidence Intervals for the ‘Severe aggression’ final model (Significant values in bold)**

| **Term** | **2.5 %** | **97.5 %** |
| --- | --- | --- |
| (Intercept) | -1,27987 | 0,542674 |
| Observer ID | -0,32545 | 1,104529 |
| Ranking | -0,1421 | 0,465625 |
| Neighbours | -0,04014 | 0,512359 |
| Friend | -0,6309 | 0,698431 |
| Sight | -0,84612 | 0,627618 |
| **Victim Call** | **0,146749** | **1,780618** |
| HR | -0,86059 | 0,68119 |
| Aggressor Call | -0,36368 | 1,193512 |
| **HR:Aggressor Call** | **0,113873** | **2,822895** |

**Supplementary Table S6. Variance Inflation Factors for each predictor from the ‘Severe aggression’ final model**

| **Observer ID** | **Ranking** | **Neighbours** | **Sight** | **AC** | **Friend** | **VC** | **HR** |
| --- | --- | --- | --- | --- | --- | --- | --- |
| 1.191897 | 1.198745 | 1.094123 | 1.179884 | 1.034988 | 1.052598 | 1.022793 | 1.276410 |
